# A Distinct Variant of Pseudohypoparathyroidism (PHP) First Characterized Some 41 Years Ago Is Caused by the 3‐kb *STX16* Deletion

**DOI:** 10.1002/jbm4.10505

**Published:** 2021-06-15

**Authors:** Zentaro Kiuchi, Monica Reyes, Arnold S Brickman, Harald Jüppner

**Affiliations:** ^1^ Endocrine Unit Massachusetts General Hospital and Harvard Medical School Boston MA USA; ^2^ School of Medicine University of California Los Angeles Los Angeles CA USA; ^3^ Pediatric Nephrology Unit Massachusetts General Hospital and Harvard Medical School Boston MA USA

**Keywords:** EPIGENETICS, GENETIC RESEARCH, PARATHYROID‐RELATED DISORDERS, DISORDERS OF CALCIUM/PHOSPHATE METABOLISM, PTH/Vit D/FGF23, CELL/TISSUE SIGNALING, ENDOCRINE PATHWAYS

## Abstract

In 1980, Farfel and colleagues (*NEJM*, 1980;303:237–42) provided first evidence for two distinct variants of pseudohypoparathyroidism (PHP) that present with hypocalcemia and impaired parathyroid hormone (PTH)‐stimulated urinary cAMP and phosphate excretion, either in the presence or absence of Albright's hereditary osteodystrophy (AHO). An “abnormal allele” and an “unexpressed allele” were considered as underlying defects, predictions that turned out to be correct for both forms of PHP. Patients affected by the first variant (now referred to as PHP1A) were later shown to be carriers of inactivating mutations involving the maternal *GNAS* exons encoding Gsα. Patients affected by the second variant (now referred to as PHP1B) were shown in the current study to carry a maternal 3‐kb *STX16* deletion, the most frequent cause of autosomal dominant PHP1B, which is associated with loss of methylation at *GNAS* exon A/B that reduces or abolishes maternal Gsα expression. However, the distinct maternal mutations leading to either PHP1A or PHP1B are disease‐causing only because paternal Gsα expression in the proximal renal tubules is silenced, ie, “unexpressed.” Our findings resolve at the molecular level carefully conducted investigations reported some 41 years ago that had provided first clues for the existence of two distinct PHP variants. © 2021 The Authors. *JBMR Plus* published by Wiley Periodicals LLC on behalf of American Society for Bone and Mineral Research.

## Introduction

In 1980, Farfel and colleagues,^(^ ^1^ ^)^ as well as Levine and colleagues,^(^ ^2^ ^)^ revealed important insights into the molecular defect responsible for a rare disease that was first described in 1942 by Albright and colleagues.^(^ ^3^ ^)^ Patients affected by this disorder present with hypocalcemia and hyperphosphatemia, in addition to unique developmental abnormalities now referred to as Albright's hereditary osteodystrophy (AHO). Even back then the authors had been able to show that hyperphosphatemia resulting from impaired urinary phosphate excretion was caused by resistance to parathyroid hormone (PTH) rather than lack of this hormone, which led to introduction of the term pseudohypoparathyroidism (PHP).^(^ ^3^ ^)^ Ten years later, Albright and colleagues furthermore described a patient with typical AHO features but without mineral ion abnormalities, and the authors thus referred to this disorder as pseudo‐pseudohypoparathyroidism (PPHP).^(^ ^4^ ^)^

No additional advances regarding the molecular mechanisms underlying both disorders were made until the discovery of cAMP as a widely used second messenger.^(^ ^5^ ^)^ This groundbreaking finding led Aurbach and colleagues to conduct experiments in which they showed that PTH stimulates cAMP production in kidney‐ and bone‐derived cell preparations.^(^ ^6^, ^7^, ^8^, ^9^ ^)^ These authors furthermore revealed that PTH dramatically increases the urinary excretion of cAMP, and with some delay the urinary excretion of phosphate, in wild‐type rats and in healthy human subjects.^(^ ^10^, ^11^ ^)^ In addition, this group of investigators reported that some PHP patients show not only a blunted PTH‐stimulated increase in cAMP excretion but also impaired phosphate excretion,^(^ ^11^ ^)^ as reported by Albright and colleagues,^(^ ^3^ ^)^ leading to the conclusion that this second messenger plays an important role in the disease pathogenesis.

**Fig 1:**
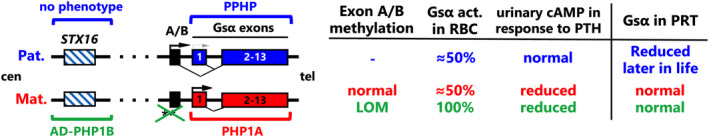
The *STX16/GNAS* complex locus depicting the location of mutations that cause PHP1A (red), PPHP (blue), and PHP1B (green) as well as laboratory and biochemical findings. Gsα (exons 1–13 of *GNAS*) is transcribed in most tissues from both parental alleles (black arrow); however, in a few tissues, including the proximal renal tubules, paternal Gsα expression is silenced (light gray arrow). Exon A/B (black box) is methylated on the maternal allele and transcription occurs only from non‐methylated allele; exons XL and AS, as well as exon NESP are not shown. Maternal Gsα mutations cause PHP1A (red bracket); paternal mutations involving these exons cause PPHP (blue bracket). Maternal deletions involving syntaxin 16 (*STX16*; box with blue strips) led to loss of methylation at *GNAS* exon A/B (green bracket and cross). Impact of the different mutations on methylation at *GNAS* exon A/B, G‐protein activity (Gsα act.) in red blood cell (RBC) membranes and on urinary cAMP excretion in response to PTH administration^(^ ^12^, ^13^ ^)^; shown is furthermore Gsα expression in normal proximal renal tubules (PRT). Boxes = exons; connecting lines = introns; Pat. = paternal; Mat. = maternal; methylated = (*); cen = centromeric; tel = telomeric. Not drawn to scale.

Subsequently, Farfel and colleagues and Levine and colleagues provided additional insights into the underlying molecular mechanism by showing, through different approaches, that some of their PHP patients, who were later classified as being affected by PHP1A, have an about 50% reduction in the activity of the guanine nucleotide regulatory protein (N‐protein or G‐unit),^(^ ^1^, ^2^ ^)^ now known to assess the α‐subunit of the stimulatory G protein (Gsα) that is encoded by *GNAS* exons 1–13 (Fig. 1). This signaling protein, which is expressed in most tissues from both parental alleles, couples a large number of diverse cell surface receptors, including the PTH/PTHrP receptor, to adenylate cyclase and thus hormone‐dependent cAMP generation.^(^ ^14^ ^)^

The reduction in peripheral blood cells to half of the normal Gsα activity was subsequently shown to be caused by heterozygous *GNAS* mutations involving one of the 13 Gsα exons.^(^ ^15^, ^16^ ^)^ It remained uncertain, however, as to why haploinsufficiency of a ubiquitously expressed signaling protein should lead to disease until Davies and Hughes showed that maternal Gsα mutations are the cause of PHP1A, whereas the same or similar mutations located on the paternal *GNAS* allele lead to PPHP.^(^ ^17^ ^)^ This critically important observation suggested that the PTH‐dependent generation of cAMP in the proximal renal tubules relies on Gsα expression from the maternal *GNAS* allele. Consistent with this conclusion, patients affected by PPHP show after PTH administration a normal increase in urinary cAMP excretion^(^ ^11^ ^)^ (Fig. 1).

Besides patients affected by PHP1A, who have only 50% of the normal G protein activity, Farfel and colleagues had also investigated 5 individuals from a single kindred who had plasma and urinary abnormalities that were indistinguishable from those of their PHP1A patients, yet these individuals showed normal Gsα activity (see Tables 1 and 2 in Farfel and colleagues^(^ ^1^ ^)^). The authors furthermore noted that the patients affected by this distinct PHP variant did not present with typical AHO features and therefore predicted a genetic defect, which differs from that responsible for PHP1A.

PTH resistance despite normal Gsα activity and without AHO features led to the hypothesis that the latter PHP variant is caused by pathogenic variants in the PTH/PTHrP receptor, which were, however, excluded through several independent approaches.^(^ ^18^, ^19^, ^20^, ^21^ ^)^ Instead, genetic linkage studies revealed that the autosomal dominant variant of PHP1B (AD‐PHP1B) is an imprinted disorder that is caused by a maternally inherited mutation centromeric of *GNAS*, which is furthermore associated with epigenetic changes at this locus.^(22‐^ ^24^ ^)^ A 3‐kb deletion was subsequently identified within *STX16*, the gene encoding syntaxin 16, which is now known to be the most frequent molecular cause of AD‐PHP1B.^(^ ^12^, ^25^, ^26^ ^)^

The carefully conducted studies by Farfel and colleagues^(^ ^1^ ^)^ had provided first evidence for a PHP variant that is distinct from PHP1A, despite indistinguishable abnormalities in the PTH‐dependent regulation of calcium and phosphate homeostasis. To determine whether the disease in their kindred E is caused by one of the known mutations or yet another familial genetic defect, we searched for the 3‐kb *STX16* deletion as the most likely underlying molecular cause.

## Materials and Methods

### Genomic DNA

We investigated genomic DNA from the four affected family members (7E–10E) described by Farfel and colleagues,^(^ ^1^ ^)^ as well as samples from 4 additional patients from the same kindred (A56, A83, A84, and A89). These samples and a preliminary drawing of the kindred had been provided by the late Dr Cornelius van Dop to help search for mutations in the PTH/PTHrP receptor that were thought at the time to cause PHP1B.^(^ ^18^ ^)^

### Multiplex PCR to search for the 3‐kb STX16 deletion

**Fig 2:**
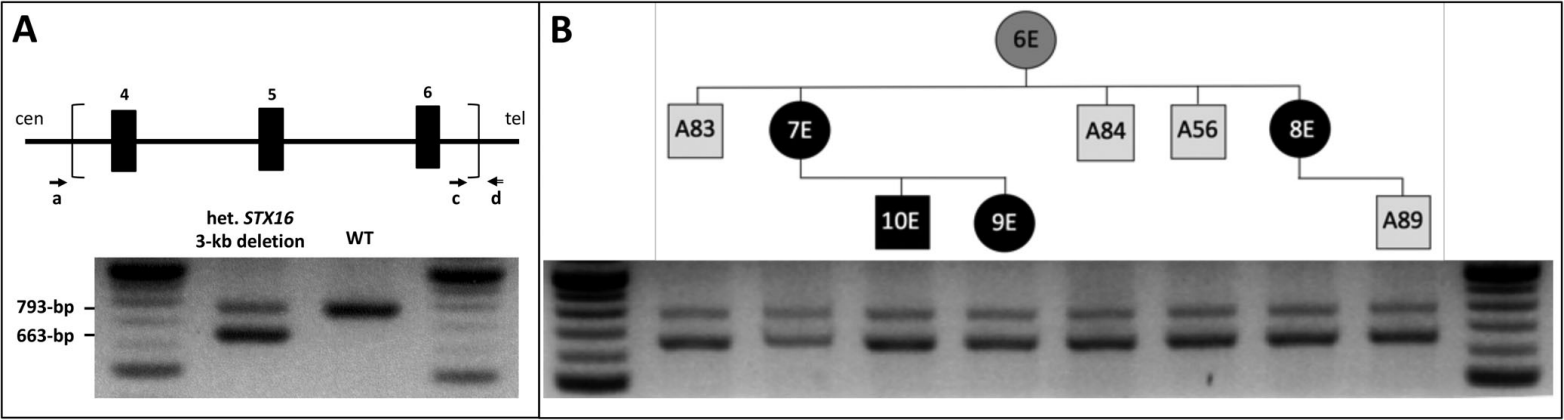
(*A*) Schematic presentation of *STX16* exons 4–6 (black boxes) and location of the PCR primers to assess the absence or presence of the frequent 3‐kb deletion, as described.^(^ ^27^ ^)^ In the presence of the 3‐kb *STX16* deletion, the portion between parentheses is missing. In that case, a 663‐bp PCR product is generated with primers a and d (when using these two primers, the PCR product derived from the wild‐type (WT) allele, ie, without the deletion, is 3641 bp in length, which is too large for amplification under the above conditions). However, portions of the normal allele are amplified using primers c and d (793 bp). Left lane: positive control for 3‐kb *STX16* deletion; right lane: normal DNA (WT). The 100‐bp ladder is shown on both sides of the gel; the prominent bands represent the 500‐ and 1000‐bp size markers. (*B*) Upper: Pedigree of the investigated kindred with autosomal dominant PHP1B (AD‐PHP1B). Black circles (females) and squares (males) with the identifiers used for the previously reported affected individuals^(^ ^1^ ^)^; dark gray circle = previously reported propositus; light gray squares = additional affected individuals. Lower: Gel electrophoresis of PCR‐amplified genomic DNA revealed that all eight affected patients are carriers of the 3‐kb *STX16* deletion; as in (*A*), upper and lower bands are derived from the normal and the mutant allele, respectively. Unfortunately, DNA was never obtained from the female 6E, thus no genetic and epigenetic studies could be performed. However, she was most likely an unaffected carrier of a paternal 3‐kb *STX16* deletion because her serum calcium and PTH levels were reported as normal.^(^ ^1^ ^)^

Multiplex amplification across *STX16* exons 4–6 to assess the absence or presence of the 3‐kb deletion was performed as described^(^ ^27^ ^)^ (Fig. 2*A*) using Qiagen Taq DNA polymerase (Qiagen, Valencia, CA, USA; Taq PCR Core Kit, catalog no./ID 201225) and three different primers (forward primers: 5′‐TTGGCAGATAACTGCTGTGG‐3′ [primer a]; 5′‐GGTGGAGCAGAACACACTGA‐3′ [primer c]; reverse primer: 5′‐CCACCTGTGGCATCATGTTA‐3′ [primer d]). Thermal cycler program: denaturation at 94°C for 5 minutes followed by 35 cycles at 94°C for 1 minute, 56°C for 1 minute, and 72°C for 1 minute, followed by a final elongation step at 72°C for 10 minutes. In the presence of the 3‐kb *STX16* deletion, a 663‐bp PCR product is generated with primers a and d that is derived from the mutant allele; a 793‐bp product derived from the wild‐type allele is amplified when using primers c and d.

### Methylation‐sensitive multiplex ligation‐dependent probe amplification (MS‐MLPA)

*GNAS* methylation changes were assessed by MLPA using kit ME031 GNAS (MRC‐Holland, Amsterdam, The Netherland; https://www.mlpa.com/), as reported.^(^ ^28^ ^)^

### Analysis of microsatellite markers

The investigated microsatellite markers in the chromosome 20q13.3 region included D20S86, 907‐rep2, 261P9‐CA, 806M20‐CA, 543J19‐TTA, and D20S171^(^ ^22^, ^23^, ^25^ ^)^; in addition, the X‐chromosomal marker DXS990 and the Y‐chromosomal marker DYS389 were analyzed to confirm the sex provided through the available records. All markers were analyzed at the Center for Human Genetic Research of the Massachusetts General Hospital, as described.^(^ ^29^ ^)^

## Results

The sex of the eight affected family members depicted in the pedigree (Fig. 2*B*) was confirmed through the analysis of markers DXS990 and DYS389, respectively (data not shown). All eight DNA samples revealed the 3‐kb deletion in *STX16*, located centromeric of *GNAS*, which is now known to be the most frequent cause of AD‐PHP1B, if located on the maternal allele.^(^ ^12^, ^30^ ^)^ Furthermore, analysis of several microsatellite markers across the *STX16/GNAS* region showed that the investigated affected individuals all share the same haplotype for the chromosome 20q13 region (data not shown).

Genomic DNA had never been obtained from the female 6E, born in 1916, thus no genetic and epigenetic studies could be performed. However, she was most likely an unaffected carrier of the 3‐kb *STX16* deletion because her serum calcium and PTH levels had been reported as normal.^(^ ^1^ ^)^ Genomic DNA from her five affected children (three males and two females) as well as three of her daughters' affected children revealed loss of methylation restricted to *GNAS* exon A/B (mean ± SD 2.0 ± 1.6%, *n* = 8; normal ≈50%), which is consistent with inheritance of the 3‐kb *STX16* deletion from a female; the other differentially methylated *GNAS* regions showed no epigenetic defect. Unfortunately, DNA had not been collected from the two unaffected children of 6E and two additional children were already deceased when molecular genetic studies were initially pursued.

## Discussion

In their insightful discussion 41 years ago, Farfel and colleagues correctly predicted that the molecular defect in their kindred E is different from that of the other PHP patients investigated in their report.^(^ ^1^ ^)^ This conclusion was based on the finding that the affected members of that family revealed normal G‐protein activity and showed no AHO features, despite hypocalcemia, hyperphosphatemia, and impaired urinary cAMP excretion in response to PTH administration, ie, biochemical findings that were indistinguishable from those observed in their PHP1A patients.^(^ ^1^ ^)^ The authors had therefore postulated that the two distinct PHP variants might be caused by different “abnormal” alleles, but they considered also the possibility of “unexpressed” alleles.

Both predictions turned out to be correct for PHP1A and PHP1B. While PHP1A is caused by heterozygous mutations involving those *GNAS* exons that encode Gsα, these inactivating genetic defects on the maternal allele lead to disease only because paternal Gsα expression is, through as‐yet undefined mechanisms, reduced or absent in some tissues, including the proximal renal tubules.^(^ ^12^, ^30^ ^)^ The maternal 3‐kb *STX16* deletion, which was now identified as the cause of PHP1B in kindred E, leads to loss of methylation at *GNAS* exon A/B, thereby reducing or abolishing maternal Gsα expression. Consequently, Gsα is “unexpressed” from both parental alleles in the portion of the kidney that is responsible for increasing 1,25(OH)_2_ vitamin D levels and for promoting urinary phosphate excretion through the PTH‐dependent formation of cAMP. The findings by Farfel and colleagues some 41 years ago thus had provided important first insights into the complex mechanisms responsible for two PHP variants that were now resolved at the genetic level.

## Disclosures

All authors state that they have no conflicts of interest.

## AUTHOR CONTRIBUTIONS

**Zentaro Kiuchi:** Conceptualization; data curation; formal analysis; methodology; writing‐original draft. **Monica Reyes:** Formal analysis; methodology; writing‐review & editing. **Arnold Brickman:** Investigation; writing‐review & editing.

### Peer review

The peer review history for this article is available at https://publons.com/publon/10.1002/jbm4.10505.
